# Acceptability of Voluntary Medical Male Circumcision (VMMC) among Male Sexually Transmitted Diseases Patients (MSTDP) in China

**DOI:** 10.1371/journal.pone.0149801

**Published:** 2016-02-23

**Authors:** Zixin Wang, Tiejian Feng, Joseph T. F. Lau, Yoona Kim

**Affiliations:** 1 Centre for Health Behaviours Research, JC School of Public Health and Primary Care, The Chinese University of Hong Kong, Hong Kong SAR, China; 2 Shenzhen Research Institute, The Chinese University of Hong Kong, Shenzhen, China; 3 Shenzhen Center for Chronic Disease Control, Shenzhen, China; 4 Centre for Medical Anthropology and Behavioral Health, Sun Yat-sen University, Guangzhou, China; Cardiff University, UNITED KINGDOM

## Abstract

Voluntary Medical Male circumcision (VMMC) is an evidence-based, yet under-utilized biomedical HIV intervention in China. No study has investigated acceptability of VMMC among male sexually transmitted diseases patients (MSTDP) who are at high risk of HIV transmission. A cross-sectional survey interviewed 350 HIV negative heterosexual MSTDP in Shenzhen, China; 12.0% (n = 42) of them were circumcised at the time of survey. When the uncircumcised participants (n = 308) were informed that VMMC could reduce the risk of HIV infection via heterosexual intercourse by 50%, the prevalence of acceptability of VMMC in the next six months was 46.1%. Adjusted for significant background variables, significant factors of acceptability of VMMC included: 1) emotional variables: the Emotional Representation Subscale (adjusted odds ratios, AOR = 1.13, 95%CI: 1.06–1.18), 2) cognitive variables derived from Health Belief Model (HBM): perceived some chance of having sex with HIV positive women in the next 12 months (AOR = 2.48, 95%CI: 1.15–5.33) (perceived susceptibility), perceived severity of STD infection (AOR = 1.06, 95%CI: 1.02–1.10), perceived benefit of VMMC in risk reduction (AOR = 1.29, 95%CI: 1.16–1.42) and sexual performance (AOR = 1.45, 95%CI: 1.26–1.71), perceived barriers against taking up VMMC (AOR = 0.88, 95%CI: 0.81–0.95), and perceived cue to action (AOR = 1.41, 95%CI: 1.23–1.61) and self-efficacy (AOR = 1.38, 95%CI: 1.26–1.35) related to taking up VMMC. The association between perceived severity of STD infection and acceptability was fully mediated by emotional representation of STD infection. The relatively low prevalence of circumcision and high acceptability suggested that the situation was favorable for implementing VMMC as a means of HIV intervention among MSTDP in China. HBM is a potential suitable framework to guide the design of future VMMC promotion. Future implementation programs should be conducted in STD clinic settings, taking the important findings of this study into account.

## Introduction

In China, an increasing number of new HIV cases have been attributed to heterosexual transmission [1]. Some sexually transmitted diseases (STD) increase the risk of HIV transmission by multi-folds [2,3]. The number of newly reported STD cases increased from 166 in 1981 to 119,955 in 2008 [4]. The, probably underestimated, HIV prevalence was 0.7% to 0.8% among male STD patients (MSTDP) in China [5,6]. Many MSTDP are clients of female sex workers (CFSW), who showed low prevalence of condom use of about 5% with their regular sex partners (RP) and about 30% with non-regular sex partners (NRP) [5,6]; they may spread HIV to lower risk female general populations.

Voluntary medical male circumcision (VMMC) is an evidence-based biomedical HIV intervention that is recommended by the World Health Organization (WHO) and the Joint United Nations Programme on HIV/AIDS (UNAIDS) for prevention of HIV-1 infections among heterosexual men [7]. Randomized trials showed that it could reduce the risk of HIV-1 infections by over 50% [8–10], prevent other types of STDs (e.g., human papillomavirus (HR-HPV) [11,12], HSV-2 [11], syphilis [13] and Trichomonas vaginalis) among heterosexual men [14], and reduce female partners’ risks of STDs [15]. Prevalence of male circumcision is below 5% among males in China [16], which is lower than that in the U.S. (75%), Republic of Korea (60%) and Philippines (90%) [17]. VMMC is a highly accessible procedure that can be performed in most of the hospitals in China. The average market rate for VMMC is about 700 RMB (about 108 USD), as compared to the mean monthly income of 5,000RMB among residents in Shenzhen [18], hence it is not excessive. Although there is no norm encouraging VMMC, there is also apparently no stigma against circumcised people. It is readily and highly available to the male general population.

Six studies were conducted on acceptability of VMMC among male heterosexual populations (general population, migrants and drug users) in China [19–24]. Five reported prevalence of acceptability of VMMC, ranging from 37.3% to 63.8%. Associated factors of acceptability of VMMC included education level [20], HIV/STD prevention service utilization [23], overly long foreskin [20,22], having had sex with NRP or FSW [20,23,24], and having a male circumcised friend [20,21]. Cognitive facilitating factors included beliefs that VMMC could improve genital hygiene [23], reduce risk of HIV/STD infection [22,23], improve sexual pleasure, increase female partners’ sexual pleasure [23–25], and lower perceived chance of HIV infection in the future [23,24]. Cognitive barriers against VMMC included perceptions that VMMC might result in sexual dysfunction [24] or infertility [23]. None of these studies used a behavioral health theory or emotional factors to explain acceptability systematically. There was only one pilot study investigating promotion of VMMC; it reported encouraging results and was also conducted among MSTDP in China [26].

Illness representation assesses how patients perceive diseases [27]. It includes cognitive and emotional representations (ER). ER is defined as the extent that patients are emotionally affected by the illness (e.g., feelings of anger, guilt or shame) [27]; it determines behaviors that may affect appraisals and health-related outcomes [28]. No study has investigated associations between STD-related ER and VMMC, which is a potential coping response of ER. Since ER is modifiable via interventions [29], it has potential implications on VMMC promotion among MSTDP. Behavioral health theories are useful in explaining health-related behaviors and guiding the design of interventions [30–32]. The Health Belief Model (HBM) consists of six constructs (i.e., perceived susceptibility, perceived severity, perceived benefit, perceived barriers, cue to action and self-efficacy) [33]. We contended that constructs related to VMMC could be potential determinants of acceptability of VMMC; such hypotheses have not been tested in previous studies. Furthermore, emotions can interact with cognitions to affect behaviors [27]. No study has investigated whether ER would moderate or mediate associations between cognitions and acceptability of VMMC.

In a sample of uncircumcised heterosexual MSTDP in Shenzhen, we investigated prevalence of acceptability of VMMC after information was given to them on HIV risk reduction and associated factors (i.e. background variables, ER and HBM-related factors). In addition, we tested the hypothesis that ER would interact with HBM-related factors to affect acceptability of VMMC (moderation effects). In case there was no moderation effect, we tested the hypothesis that ER and HBM factors would have independent associations with acceptability of VMMC (mediation effect).

## Materials and Methods

### Study Participants and Data Collection

Participants were recruited from the three largest governmental STD clinics in Shenzhen, China during October 2011 to March 2012. Inclusion criteria were: 1) Chinese male ≥ 18 years old and 2) diagnosis of any one of the five types of STD listed in the national surveillance system [34]: Primary, secondary or latent syphilis [determined by Treponemapallidum Particle Assay (TPPA) and Toluidine Red Untreated Serum Test (TRUST)], genital warts (diagnosed by presence of clinical symptoms and supported by biopsy), genital herpes (diagnosed clinically, supported by ELISA), or Gonorrhea/NGU (diagnosed by using Polymerase Chain Reaction). Men who have ever had oral or anal sex with men (MSM) and those who were known to be HIV positive were excluded from the study, as efficacy of VMMC in reducing HIV transmission is only available for HIV negative heterosexual men but not for MSM or HIV positive men [35,36]. Also, WHO recommendation for VMMC is for HIV negative heterosexual men but not MSM or HIV positive men [7].

To avoid selection bias, we invited all male STD patients attending the participating clinics during the study period to participate in the study. A clinician examined the foreskin condition of the prospective participant and ensured his eligibility to join the study in a consultation room. Participants were assured that refusals would not affect their right to use any services and they could quit any time without being questioned. A total of 362 eligible MSTDP were approached by the interviewers; 12 (3.3%) declined to participate in the study, 350 (96.7%) provided written informed consent and completed an anonymous face-to-face interview using a structured questionnaire, which took about 30 minutes to complete. A monetary compensation (50 RMB or ~8 USD) was given to the participants upon completion of the interview for their time spent. Ethics approval was obtained from the Survey and Behavioural Research Ethics Committee, the Chinese University of Hong Kong. Signed informed consent was obtained from each participant in the first encounter.

### Measures

A panel was formed to design the questionnaire. It consisted of two STD clinicians, two HIV/AIDS epidemiologists and one psychologist. Discussion was made to create and finalize the questionnaire. The preliminary questionnaire was tested among six MSTDP before its finalization.

Foreskin conditions of the uncircumcised participants were recorded by clinicians in three categories: 1) glans completely exposed in the absence of an erection, 2) glans partially covered by foreskin in the absence of an erection and 3) foreskin completely covering glans in the absence of an erection but can be retracted properly, which was defined as overly long foreskin [37].

Uncircumcised participants were asked whether they were willing to take up VMMC in the next six months after having been informed that it could reduce risk of HIV infection via heterosexual intercourse by 50%.

Participants were asked about their socio-demographic information, HIV/STD-related knowledge, HIV/STD-related services utilization and sexual behaviors (see Table 1 for all items). The 6-item validated Emotional Representation Subscale of the Revised Illness Perception Questionnaire (IPQ-R) of STD infection (IPQ-R-STD, available at http://www.uib.no/ipq/pdf/IPQ-R-STD.pdf), was used to assess emotional representation of STD. The subscale was translated into Chinese by a panel of two bilingual psychologists and was back translated into English by another two bilingual epidemiologists who were blinded to the original scale. An example was, “I get depressed when I think about my STD” (see Table 2 for all items). Exploratory factor analysis (EFA) found that the items made up one factor which explained 61.3% of the total variance (Cronbach’s alpha = 0.867).

**Table 1:** Background characteristics of the respondents.

	All (n = 350)%	Uncircumcised (n = 308) %	Circumcised (n = 42) %	P value
**Socio-demographic variables**				
Age group				
18–30	37.7	37.7	38.1	
31–40	36.9	37.3	33.3	
41–50	18.9	18.2	23.8	
>50	6.6	6.8	4.8	0.796
Current marital status				
Single	28.3	29.2	21.4	
Married or cohabited with a woman	66.9	65.6	76.2	
Divorced, separated or widowed	4.9	5.2	2.4	0.365
City of permanent residence (Hukou)				
Shenzhen	29.1	28.9	31.0	
Other city in Guangdong	21.7	22.4	16.7	
Other province in China	49.1	48.7	52.4	0.699
Highest education level attained				
Junior high or lower	32.3	33.1	26.2	
Senior high school (or equal academic qualification)	27.1	26.9	28.6	
College	19.7	18.5	28.6	
University or above	20.9	21.4	16.7	0.410
Monthly personal income (RMB)				
<3,000	22.9	23.1	21.4	
3,000–4,999	28.9	28.9	28.6	
5,000–9,999	18.9	18.5	21.4	
≥ 10,000	29.4	29.5	28.6	0.974
**HIV/STD-related knowledge (correct response)**				
People with HIV positive can look healthy	56.9	56.5	59.5	0.710
HIV antibody cannot be detected one week after HIV transmission	25.4	24.7	31.0	0.381
People can contract the same STD more than once	56.9	55.2	69.0	0.089
STD infection will increase the risk of contracting HIV	40.3	39.6	45.2	0.485
Number of correct responses				
0	25.7	27.6	11.9	
1–2	39.7	38.4	50.0	
3–4	34.6	34.0	38.1	0.083
**HIV/STD-related services utilized in the last 6 months**				
HIV antibody testing	37.7	37.3	40.5	0.694
Condom distribution	12.3	12.7	9.5	0.561
Peer education	8.3	8.1	9.5	0.756
Lecture on HIV/STD knowledge	7.7	8.1	4.8	0.445
HIV/STD pamphlet	24.6	25.6	16.7	0.205
**Foreskin conditions**				
Glans completely exposing in the absence of erection	---	18.2	---	---
Foreskin partially covered glans in the absence of erection	---	43.5	---	---
Foreskin completely covered glans in the absence of erection, but can be retracted properly (overly long foreskin)	---	38.3	---	---
**STD history**				
Current STD infection				
Primary syphilis	3.4	3.6	2.4	
Secondary syphilis	2.6	2.6	2.4	
Latent syphilis	17.1	17.5	14.3	
Genital warts	40.9	39.0	54.8	
Genital herpes	15.7	16.2	11.9	
Gonorrhea or NGU	20.3	21.1	14.3	0.559
Episodes of STD infection in previous 3 years				
1	73.7	73.1	78.6	
2	17.7	18.5	11.9	
≥3	8.6	8.4	9.5	0.573
**Sexual behavior in the last six months**				
Number of Regular female sex partners (RP) with sexual intercourse				
0	20.3	21.4	11.9	
1	76.6	75.6	83.3	
≥2	3.1	2.9	4.8	0.311
Number of non-regular female sex partners (NRP) with sexual intercourse				
0	62.0	61.0	69.0	
1	22.3	22.4	21.4	
≥2	15.7	16.6	9.5	0.458
Had patronized FSW				
No	57.1	57.1	57.1	
Yes	42.9	42.9	42.9	1.000
**Sexual behaviors since exhibiting STD symptoms or receiving an STD diagnosis**				
Had had unprotected sex with RP				
No/had not had sex with RP	76.0	75.6	78.6	
Yes	24.0	24.4	21.4	0.667
Had had unprotected sex with NRP				
No/had not had sex with NRP	91.1	90.6	95.2	
Yes	8.9	9.4	4.8	0.319
Had had unprotected sex with FSW				
No/had not had sex with FSW	92.0	91.9	92.9	
Yes	8.0	8.1	7.1	0.827

Regular female sex partner(s) (RP): wife or girlfriend

Female sex worker (FSW): female who exchanged sex for money.

Non-regular female sex partner(s) (NRP): female who were neither RP (wife or girlfriend) nor FSW.

**Table 2:** Frequency distribution of cognitive variables derived from HBM and emotional representation of STD infection among uncircumcised male STD patients (n = 308).

	% / Mean (SD)
**Emotional Representation of STD infection** (% strongly agree/agree)	
I get depressed when I think about my STD	63.0
When I think about my STD, I get upset	65.9
My STD makes me feel angry	47.1
My STD does not worry me	36.4
Having this STD make me feel anxious	60.1
My STD makes me feel afraid	59.1
Scale Score	
Emotional Representation Subscale	19.5 (5.2)
**HBM-related factors**	
**Perceived susceptibility**	
Do you perceive some chance of having sex with HIV positive women in the next twelve months	
No	86.4
Yes	13.6
**Perceived severity**	
Perceived severity of HIV infection (% Severe/very severe)	
Harm of HIV infection on physical Health	92.5
Harm of HIV infection on mental health	92.5
Harm of HIV infection on work	84.7
Harm of HIV infection on personal economic status	85.1
Harm of HIV infection on family relationships	90.6
Harm of HIV infection on social life	87.7
Scale Score	
HIV Perceived Severity Scale	26.8 (5.1)
Perceived severity of STD infection (% Severe/very severe)	
Harm of STD infection on physical Health	44.8
Harm of STD infection on mental health	52.9
Harm of STD infection on work	26.3
Harm of STD infection on personal economic status	27.9
Harm of STD infection on family relationships	27.3
Harm of STD infection on social life	21.8
Scale Score	
STD Perceived Severity Scale	15.3 (6.5)
**Perceived benefit of VMMC**	
Perceived risk reduction due to VMMC (% strongly agree/agree)	
VMMC could reduce the risk of HIV infection	29.5
VMMC could reduce the risk of STD infection	34.4
VMMC could reduce female sex partner’s risk of gynecological diseases	48.1
VMMC could reduce female sex partner’s risk of STD infection	46.8
Scale Score	
Perceived Risk Reduction Scale	12.8 (2.8)
Perceived benefit for sexual performance (% strongly agree/agree)	
VMMC would increase sexual pleasure	22.4
VMMC would increase erectile duration	49.7
VMMC would increase female sexual partner’s sexual pleasure	19.2
Scale Score	
Sexual Performance Scale	9.1 (1.9)
**Perceived barriers against taking up VMMC** (% strongly agree/agree)	
VMMC is expensive for me	9.7
VMMC would cause pain on penis	35.1
VMMC would result in severe surgical complications (e.g. bleeding, edema and inflammation)	32.1
VMMC would result in erectile dysfunction	26.9
My peers would consider it strange for me to take up VMMC	13.0
My female sex partners would consider it strange for me to take up VMMC	10.8
I am embarrassed to take up VMMC	20.7
Scale Score	
Perceived Barrier Scale	18.5 (3.3)
**Cue to Action for taking up VMMC** (% strongly agree/agree)	
Some doctors suggested that I should take up VMMC	28.2
Some of my peers suggested that I should take up VMMC	11.4
My female sex partner suggested that I should take up VMMC	9.7
Scale Score	
Cue to Action Scale	9.1 (2.6)
**Self-efficacy for taking up VMMC** (% strongly agree/agree)	
I am confident that I can take up VMMC if desired	60.1
I am confident that I can take up VMMC in the next six months	31.2
Whether to take up VMMC or not is completely under my control	31.1
I have sufficient resources enabling me to take up VMMC in the next six months	33.8
Scale Score	
Self-efficacy Scale	12.6 (3.4)

Seven constructed scales were based on the HBM: 1) the 6-item HIV Perceived Severity Scale and the 6-item STD Perceived Severity Scale (e.g., Perceived harm of HIV infection and STD on their physical health); 2) the 4-item Perceived Risk Reduction Scale (e.g., VMMC could reduce the risk of HIV infection) and the 3-item Sexual Performance Scale (e.g., VMMC would increase sexual pleasure); 3) the 7-item Perceived Barriers Scale (e.g., VMMC would cause pain on penis); 4) the 3-item Cue to Action Scale (e.g., Some doctors suggested that I should take up VMMC) and 5) the 4-item Self-efficacy Scale, (e.g., I am confident that I can take up VMMC if desired) (Response categories: 1 = strongly disagree, 5 = strongly agree). Cronbach’s alpha of these seven scales were acceptable (0.779~0.944); single factors were identified for these seven scales by using EFA, explaining 64.9% to 96.3% of the total variances. The item “Do you perceive some chance of having sex with HIV positive women in the next twelve months” was also asked (perceived susceptibility) (Response categories: 0 = no, 1 = yes). All scale items were listed in Table 2.

### Statistical Analysis

Prevalence of VMMC among all MSTDP and prevalence of acceptability of VMMC among uncircumcised participants were presented. Using acceptability of VMMC as the dependent variable, univariate odds ratios (ORu) of the background variables were identified. Multivariate odds ratios (ORm) were obtained by fitting multiple forward stepwise logistic regression models using the significant background variables as candidates for selection. Adjusted for significant background variables, adjusted odds ratios (AOR) for the associations between independent variables of interest (emotional variables and HBM-related variables) and the dependent variable were derived by fitting multiple logistic regression models. Similar analyses have been performed in numerous published studies [38–40].

To test the moderation hypotheses, six multivariate logistic regression models, each adjusted for background variables, were fit, and contained a main effect variable (e.g., STD Perceived Severity Scale) and its interaction term with ER status (higher or lower ER status using 50^th^ percentile of the Emotional Representation Scale using as cut-off point). Interaction effects were tested by comparing the model containing the main effect variables against those both main effect variables and their interaction term, using the change in -2Log (likelihood) (-2LL) statistics.

In the absence of significant interaction effects, the mediation effect hypothesis was tested following Baron and Kenny’ method [41]. For a significant mediation effect to occur, the HBM-related variables should have significant associations with both the Emotional Representation Subscale (the mediator) and acceptability of VMMC (the dependent variables). If the association between the HBM-related variables and acceptability of VMMC became statistically non-significant after adjusting for the Emotional Representation Subscale then, the Emotional Representation Subscale was seen to mediate the association between the HBM-related variables and acceptability of VMMC fully. The SPSS version 16.0 was used for data analysis, with *p* values < .05 taken as statistically significant.

## Results

### Descriptive Statistics

Participants’ STD diagnosis was: primary syphilis (3.4%), secondary syphilis (2.6%), latent syphilis (17.1%), genital warts (40.9%), genital herpes (15.9%), and gonorrhea/non-gonococcal urethritis (20.3%). No one was infected with ≥2 types of STDs at the same time and 26.3% had had multiple episodes of STD infections in the last three years. The majority of the participants were ≤40 years old (74.6%), married or cohabiting with a female partner (66.9%) and not a permanent resident of Shenzhen (70.8%). Of the participants, 40.6% had attended colleges or universities and 29.4% had a monthly income of 10,000 RMB (US$ 1600) or more. No difference in background variables was observed when comparing uncircumcised MSTDP to those who had already been circumcised (42/350, 12.0%, 95% CI: 8.6%~15.4%). (Table 1)

Among uncircumcised MSTDP, 38.3% had overly long foreskin. The prevalence of acceptability of VMMC in the next six months was 46.1% (95% CI: 40.5%~51.7%) after having been informed that it could reduce risk of HIV infection.

### ER of STD Infection

Half or more agreed that their STD made them feel depressed (63.0%), upset (65.9%), anxious (60.1%), afraid (59.1%) or angry (47.1%), while only 36.4% did not worry about their STD. (Table 2).

### Cognitive Variables Derived from the HBM

Only a minority (13.6%) of the participants perceived some chance in having sex with HIV positive women within the next 12 months (i.e. perceived susceptibility). The majority perceived that HIV infection would cause severe or very severe harms to their physical health, mental health, work, finance, family relationship and social activity (84.7% to 92.5%), while fewer perceived that STD infection would have such severe or very severe harms (21.8% to 52.9%). Less than half perceived that VMMC has some benefits, such as could reduce their risk of HIV infection (29.5%) or STD infection (34.4%), reduce their female sex partner’s risk of gynecological diseases (48.1%) or STD infection (46.8%), increase their sexual pleasure (22.4%) or erectile duration (49.7%), or increase their female partner’s sexual pleasure (19.2%). Not many participants perceived barriers against taking up VMMC, including the beliefs that uptake of VMMC was expensive (9.7%), would cause pain on the penis (35.1%), would result in severe surgical complications (32.1%) or erectile dysfunction (26.9%), would be perceived as strange by their peers (13.0%) or their female sex partners (10.8%), or would be embarrassing (20.7%). Only a minority had received cue to action about VMMC from doctors (28.2%), peers (11.4%) or female sex partners (9.7%). The level of perceived self-efficacy was moderate as 31.1% to 60.1% agreed or strongly agreed with the four related statements. (Table 2)

### Associations between Background Variables and Acceptability of VMMC after Having Been Informed that It Could Reduce Risk of HIV Infection

In the multivariate analysis, four background variables were significantly associated with acceptability of VMMC after having been informed that it could reduce the risk of HIV infection: 1) aged > 40 years old (ORm = 0.39, 95% CI: 0.21–0.72), 2) HIV/STD-related knowledge (3~4 correct responses, ORm = 2.61, 95% CI: 1.35–5.03; 1~2 correct responses, ORm = 1.97, 95% CI: 1.05–3.69; reference group: 0 correct response), 3) overly long foreskin (ORm = 3.31, 95% CI: 1.63–6.73; reference group: glans completely exposed in the absence of erection) and 4) having contracted three or more episodes of STD in the last three years (ORm = 4.78, 95% CI: 1.73–13.21; reference group: only this episode) (Table 3). These factors were adjusted for in subsequent analysis. Types of STD infection was not significantly associated with acceptability of VMMC (p = .354, Chi-square test).

**Table 3:** Association between background factors and acceptability of VMMC after having been informed that it could reduce risk of HIV infection.

	Row%	ORu (95%CI)	ORm (95%CI)
**Socio-demographic variables**			
Age group			
18–40	50.6	1.0	1.0
>40	32.5	0.47 (0.27–0.81)**	0.39 (0.21–0.72)**
City of permanent residence (Hukou)			
Shenzhen	53.9	1.0	
Other cities in China	42.9	0.64 (0.39–1.05) †	NS
**HIV/STD-related knowledge**			
Number of correct responses ^1^			
0	29.4	1.0	1.0
1–2	48.3	2.24 (1.24–4.05)**	1.97 (1.05–3.69)*
3–4	57.1	3.20 (1.75–5.87)***	2.61 (1.35–5.03)**
**HIV/STD-related services utilized**			
HIV antibody testing (last year)			
No	42.5	1.0	
Yes	52.2	1.47 (0.93–2.34)†	NS
Condom distribution (last 6 months)			
No	43.5	1.0	
Yes	64.1	2.32 (1.16–4.66)*	NS
Peer education (last 6 months)			
No	44.5	1.0	
Yes	64.0	2.22 (0.95–5.18)†	NS
**Foreskin conditions**			
Glans completely exposing in the absence of erection	35.7	1.0	1.0
Foreskin partially covering glans in the absence of erection	36.6	1.04 (0.54–1.99)	1.07 (0.54–2.13)
Foreskin completely covering glans in the absence of erection, but can be retracted properly (Overly long foreskin)	61.9	2.92 (1.51–5.66)**	3.31 (1.63–6.73)**
**STD history**			
Episodes of STD infection in last 3 years			
1	40.0	1.0	1.0
2	56.1	1.92 (1.07–3.45)*	1.82 (0.99–3.57)†
≥3	76.9	5.00 (1.93–12.94)**	4.78 (1.73–13.21)**
**Sexual behaviors in the last six months**			
Number of non-regular female sex partners (NRP) with sexual intercourse			
0	41.0	1.0	
1	46.4	1.25 (0.72–2.17)	
≥2	64.7	2.64 (1.39–5.03)**	NS

ORu: univariate odds ratios; ORm: multivariate odds ratios obtained from stepwise logistic regression using background variables P<0.10 in univariate analysis as candidates (entry: P<0.10; exclude: P>0.20) NS: P>0.10; --: univariately non-significant not considered in the model

† P<0.10

* P<0.05

** P<0.01

*** P<0.001

^1^ Number of correct responses to four HIV/STD related knowledge items: 1) People with HIV positive can look healthy; 2) HIV antibody cannot be detected 1 week after HIV transmission; 3) People can contract same STD more than once and 4) STD infection will increase the risk of contracting HIV.

### Associations between Cognitive Factors, ER and Acceptability of VMMC after Having Been Informed that It Could Reduce Risk of HIV Infection

Adjusted for these four background variables, significant emotional and HBM–related variables were: 1) the Emotional Representation Subscale (AOR = 1.13, 95% CI: 1.06–1.18), 2) perceiving some chance in having sex with HIV positive women in the next 12 months (AOR = 2.48, 95% CI: 1.15~5.33) (perceived susceptibility), 3) The STD Perceived Severity Scale (AOR = 1.06. 95% CI: 1.02–1.10), 4) The Perceived Risk Reduction Scale (AOR = 1.29, 95% CI: 1.16–1.42) (perceived benefit), 5) The Sexual Performance Scale (AOR = 1.45, 95% CI: 1.26–1.71) (perceived benefit), 6) The Perceived Barrier Scale (AOR = 0.88, 95% CI: 0.81–0.95), 7) The Cue to Action Scale (AOR = 1.41, 95% CI: 1.23–1.61), and 8) The Self-efficacy Scale (AOR = 1.38, 95%CI: 1.26–1.52). (Table 4)

**Table 4:** Associations between HBM-related variables, emotional representation of STD infection and acceptability of VMMC after having been informed that it could reduce risk of HIV infection.

	ORu (95%CI)	AOR (95%CI)
**Emotional representation of STD infection**		
Emotional Representation Subscale	1.12 (1.07–1.18)***	1.13 (1.06–1.18)***
**HBM related variable**		
Perceived susceptibility		
Do you perceive some chance of having sex with HIV positive women in the next twelve months		
No	1.0	1.0
Yes	3.02 (1.50–6.07)**	2.48 (1.15–5.33)*
Perceived severity		
STD Perceived Severity Scale	1.06 (1.02–1.10)**	1.06 (1.02–1.10)**
Perceived benefit of VMMC		
Perceived Risk Reduction Scale	1.31 (1.20–1.44)***	1.29 (1.16–1.42)***
Sexual Performance Scale	1.45 (1.26–1.67)***	1.47 (1.26–1.71)***
Perceived barriers against taking up VMMC		
Perceived Barrier Scale	0.89 (0.83–0.95)**	0.88 (0.81–0.95)**
Cue to action for VMMC uptake		
Cue to Action Scale	1.50 (1.31–1.71)***	1.41 (1.23–1.61)***
Self-efficacy for VMMC uptake		
Self-efficacy Scale	1.38 (1.26–1.50)***	1.38 (1.26–1.52)***

* P<0.05

** P<0.01

*** P<0.001

ORu: univariate odds ratio; AOR: adjusted odds ratio, odds ratios adjusted by multivariately significant background variables (listed in Table 1)

### Testing the Moderation Hypotheses

None of the interactive terms between ER and cognitive variables with acceptability of VMMC as the dependent variable were found to be statistically significant (Δ-2LL = 0.012–3.459, df = 1, *p* > .05, chi-square test; data not tabulated). No moderation effect was hence detected.

### Testing the Mediation Hypothesis

The results of the linear regression analysis showed that the STD Perceived Severity Scale (B = 0.408, S.E. = 0.040, *p* < .001), the Perceived Risk Reduction Scale (B = 0.380, S.E. = 0.103, P < .001), the Cue to Action Scale (B = 0.273, S.E. = 0.114, *p* = .018) and the Self-efficacy Scale (B = 0.327, S.E. = 0.085, *p* < .001) were all significantly correlated with the Emotional Representation Subscale (data not tabulated).

Adjusted for the significant background variables and in the presence of the Emotional Representation Scale, the STD Perceived Severity Scale which was of statistical significance, became statistically non-significant (p = 0.273). A fully mediating effect was hence observed. However, the other scales of the HBM remained significantly associated with acceptability of VMMC in the presence of the ER Subscale, showing independent effects existed and hence no mediating effect was demonstrated for these variables of the HBM. (Table 5)

**Table 5:** Testing for independent effect of cognitive variables and emotional representation of STD infection on the associations with acceptability of VMMC after having been informed that it could reduce risk of HIV infection.

Model	Variables	B	S.E.	Adjusted OR (95%CI) ^1^	P value
1	Emotional Representation Subscale	0.102	0.030	**1.11 (1.04–1.18)**	**0.001**
	STD Perceived Severity Scale	0.023	0.024	1.02 (0.98–1.07)	0.340
2	Emotional Representation Scale	0.101	0.029	**1.11 (1.05–1.17)**	**<0.001**
	Perceived Risk Reduction Scale	0.230	0.052	**1.26 (1.14–1.39)**	**<0.001**
3	Emotional Representation Scale	0.109	0.029	**1.12 (1.05–1.18)**	**<0.001**
	Cue to Action Scale	0.326	0.068	**1.39 (1.21–1.58)**	**<0.001**
4	Emotional Representation Scale	0.097	0.030	**1.10 (1.04–1.17)**	**0.001**
	Self-efficacy Scale	0.311	0.049	**1.37 (1.24–1.50)**	**<0.001**

^1^Adjusted logistic regression: the analysis adjusted for the multivariately significant background variable (listed in Table 3)

## Discussion

We found that 12% of the sampled MSTDP were circumcised at the time of survey. Such prevalence is comparable with that of other studies conducted in China [19,24,42]. It is much lower than that of the U.S., Republic of Korea and Philippines [17]. In China, MC is not performed as a ritual, except among some Muslim minorities which make up <2% of the country’s population [43]. In the Han majority population in China, no cultural norm exists for routine MC among adults or neonates. The relatively low prevalence further suggests that there is room for promoting VMMC as a means of HIV prevention.

To our knowledge, this is the first study investigating acceptability of VMMC among MSTDP, applying behavioral health models and ER to investigate associated factors, and studying related moderation/mediation effects. Although, there was a totally independent pilot study investigating feasibility of promoting VMMC among MSTDP without investigating prevalence and associated factors of VMMC acceptability [26]. In this study, we gauged acceptability of VMMC under the condition that its efficacy was made known to the participants, instead of acceptability in the absence of such information. In literature, it is very common for acceptability studies to explain benefits (and harms) of new biomedical preventive measures [24,44–46] to participants. The process ensures that uniform information has been received by all participants, and hence allows for better interpretation of the response related to acceptability of a new preventive measure. The observed high acceptability of VMMC provided insights for designing future VMMC promotion programs.

Other important insights focus on segmentation. According to social marketing approaches, careful segmentation would improve effectiveness [47]. First, we found that younger MSTDP were more responsive to VMMC, as they showed higher acceptability of VMMC than older MSTDP. This was understandable as younger people are usually more receptive to innovations [48]. Promotion of VMMC among different age groups may consider different strategies. Another potential segmentation criterion was based on foreskin length. STD clinicians should pay attention to MSTDP’s foreskin length and recommend those with overly long foreskins to take up VMMC; as such patients tend to have higher acceptability of VMMC. However, STD clinicians should tell those without overly long foreskin about the evidence that HIV risk reduction effect of VMMC also applies to them [49]. In addition, special attention should be given by STD clinicians to those MSTDP who had multiple episodes of STD infection, as such patients were found in this study to show significantly higher acceptability of VMMC. They might have perceived a stronger need for taking up protective measures against HIV/STD. STD clinicians should make use of the opportunity to remind such patients of their high risk of STD infection and recurrence in the future. Moreover, they should explain to such patients that many STD, such as syphilis and genital herpes infection, would increase the risk of HIV sero-conversion [2,3], and confirm that VMMC is effective in preventing heterosexual transmission of HIV/STD [8–14].

It is important to create cognitive changes to increase acceptability of VMMC. We found that knowledge on HIV/STD was significantly associated with acceptability of VMMC; it is potentially useful to improve such knowledge. However, supply of knowledge alone is not enough; interventions are warranted to change theory-based perceptions related to VMMC as well. To our knowledge, no implementation study of VMMC has been based on behavioral health theories, although applications of such theories have been found in effective HIV intervention programs [32]. We showed that the HBM is a potentially suitable framework to guide the design of future interventions on VMMC targeting MSTDP, as all of its constructs were significantly associated with acceptability of VMMC. Specifically, we should increase perceived susceptibility and perceived severity related to STD, as these two factors were significantly associated with acceptability of VMMC as well as other health behaviors [33]. Besides information dissemination, other approaches are warranted. Arousal of emotions, such as the fear appeal approach [50], is a potentially useful strategy. According to the fear appeal approach of the Extended Parallel Process model [50], perceived threat is triggered only in the presence of both perceived susceptibility and perceived severity. In our case, perceived severity of STD, but not that of HIV infection, was significantly associated with acceptability of VMMC, suggesting that arousal of fear should focus on STD rather than HIV when promoting VMMC among MSTDP. Perceived severity was non-significant, as HIV might have sounded remote to the participants because the prevalence of HIV among heterosexuals in Shenzhen remained low (about 1 in 100,000) [51]. In contrast, STD was highly relevant to them as all of them had had first-hand experience.

STD infection brings emotions, whereas cognitive theories do not take emotions into account. No previous acceptability study on VMMC has taken emotional factors into account. Importance of emotions is further illustrated by the significant association between ER and acceptability of VMMC in the adjusted analysis. We cannot increase acceptability of VMMC by increasing ER, which would be unethical. However, the results inform us that positive, instead of negative coping resulted from ER, which is encouraging. We can hence guide those with high levels of ER to take up VMMC as a positive coping response and as a preventive measure, hence transferring their emotions into positive coping and preventive behaviors.

Risk reduction was one of the perceived benefits of VMMC, another construct of the HBM, which was significantly associated with acceptability of VMMC. However, over half of the participants failed to believe in the HIV/STD risk reduction effects, even after they had been informed about specific HIV/STD risk reduction effects before they answered the questions on perceived benefit. The lesson learned is that health workers cannot take it for granted that scientific evidences will be translated perfectly into cognitions once they are disseminated to targets of health promotion. Again, efforts beyond information dissemination are required. Motivational interviewing, a client-centered counseling technique, is a potentially useful means [52].

We found that perceived benefit on improvement of sexual performance (perceived benefit) and concern about side effects (perceived barrier) were significantly associated with acceptability of VMMC. Previous studies also showed significant associations between perceived side-effect and acceptability of VMMC [23,24,53,54]. Testimonials made by peers who had taken up VMMC on potential positive effects of VMMC for their sexual performance and mildness of side effects are potentially useful.

Embarrassment and perceived stigma related to VMMC were other perceived barriers. In general, such factors were obstacles preventing sex-related health service seeking [55]. STD was also strongly associated with social stigma [56]. MSTDP might associate the uptake of VMMC with allegations of promiscuity and stigma. STD clinics then offer ideal settings for promoting VMMC which reduce embarrassment to discuss about HIV/STD. Capacity building is needed as previous studies have reported substantial stigma toward HIV-related vulnerable groups in China, even in clinical settings [57]. In contrast, only a minority of the participants perceived that high cost was involved for taking up VMMC. Therefore, financial subsidy for MSTDP to take up VMMC seems unnecessary, making future VMMC implementation programs more sustainable.

In the present study, cue to action and perceived self-efficacy were two other constructs of the HBM that were significantly associated with VMMC. However, we found that only a minority of the sampled MSTDP had been suggested by clinicians, their female sex partners or peers to take up VMMC. It is possible that VMMC is a new preventive measure and some STD clinicians in China are not familiar with the evidence. Social marketing, therefore, should not be limited to MSTDP, but also extended to target STD clinicians and female sex partners of MSTDP; there is huge room for improvement. It supports the contention that STD clinicians would be able to serve as strong cue to action and that health promotion of VMMC in STD clinics would be effective. Multiple strategies are required to improve perceived self-efficacy related to VMMC. These include counseling to reduce perceived barriers, simplification of logistics in booking operations, provision of a list of credible hospitals for operation and clear goal setting. Setting up a hotline to answer questions and facilitate appointments may be considered.

Our results did not show that ER amplified nor diminished (moderated) the associations between the constructs of the HBM and acceptability of VMMC. Of all possible mediation effects, we only found that the association between perceived severity and acceptability of VMMC was mediated by ER. Therefore, cognitive factors such as those of the HBM and emotions such as ER may largely have independent and significant effects on acceptability of VMMC, implying that similar cognitive interventions for enhancement of acceptability of VMMC can be provided to MSTDP of both high and low levels of ER. The aforementioned mediation effect for perceived severity was understandable as those who perceived severity of STD would be more likely to respond emotionally (a higher ER level) to their STD and were more eager to take up protective measures (VMMC). Caution should be made if interventions are to increase perceived severity as it might also increase ER; psychological support should hence be made available.

Although this study had some strengths such as testing a set of new hypotheses (e.g., those involving ER), it also had some limitations. First, the participants were recruited not by probability sampling, nor by respondent-driven sampling methods [58]. However, studies using a similar study design are common [20–22]. Second, we did not collect information from participants who refused to participate in the study. Thirdly, we only included patients with the five most prevalent types of STD. Although patients infected with other types of STD (e.g. chancroid) were low in number, they might also benefit from VMMC. Fourth, it is a limitation of the study that a binary dependent variable of acceptability was used, while acceptability could be considered a continuous variable. Similar binary outcomes have commonly been used in a number of published acceptability studies [24,42,45,59]. It is warranted to develop continuous scales for acceptability of VMMC in the future. Stages of change of the Trans-theoretical model [60] are commonly used to study behavioral change, and may also be applied to future studies investigating acceptability of VMMC. Moreover, self-reporting responses might have reporting bias, although anonymity should have reduced the bias. For instance, MSM might not disclose such status or participants might not like to report that they are going to have HIV positive female sex partners as they do not want to be perceived as promiscuous. VMMC is also not a norm in China and taking up VMMC is not socially desirable. Furthermore, in the absence of validated scales, the cognitive variables were self-constructed but we checked some psychometric properties such as internal reliability. Lastly, we only obtained cross-sectional associations and could not establish causal relationships. Acceptability may not be translated into actually VMMC uptake.

This formative study can be seen as a needs assessment, which is the first required step in planning effective health promotion campaigns [61]. The situation among MSTDP in China was favorable for implementing such programs as acceptability of VMMC among MSTDP was high, even in the absence of previous health promotion and when MSTDP were being introduced briefly as a risk reduction measure. The results showed that cognitive perceptions and emotional factors were both significant, and largely independent, factors of acceptability of VMMC. The constructs of the HBM form a promising theoretical framework for promoting VMMC among MSTDP. Furthermore, the STD clinic based setting approach seems appropriate, as STD clinicians induce strong cue to action but potentially lower stigma and embarrassment to their patients. Future implementation programs should be conducted, taking the important findings of this study into account.
